# The periodic table of ribonucleotide reductases

**DOI:** 10.1016/j.jbc.2021.101137

**Published:** 2021-08-27

**Authors:** Terry B. Ruskoski, Amie K. Boal

**Affiliations:** 1 Department of Biochemistry and Molecular Biology, The Pennsylvania State University, University Park, Pennsylvania, USA; 2 Department of Chemistry, The Pennsylvania State University, University Park, Pennsylvania, USA

**Keywords:** electron transfer, enzyme structure, iron, manganese, radical, Cys, cysteine, DOPA, dihydroxyphenylalanine, EPR, electron paramagnetic resonance, HU, hydroxyurea, Lys, lysine, PCET, proton-coupled electron transfer, RNR, ribonucleotide reductase, RT, radical translocation, Trp, tryptophan, Tyr, tyrosine, Tyr•, tyrosyl radical, XAS, X-ray absorption spectroscopy

## Abstract

In most organisms, transition metal ions are necessary cofactors of ribonucleotide reductase (RNR), the enzyme responsible for biosynthesis of the 2′-deoxynucleotide building blocks of DNA. The metal ion generates an oxidant for an active site cysteine (Cys), yielding a thiyl radical that is necessary for initiation of catalysis in all RNRs. Class I enzymes, widespread in eukaryotes and aerobic microbes, share a common requirement for dioxygen in assembly of the active Cys oxidant and a unique quaternary structure, in which the metallo- or radical-cofactor is found in a separate subunit, β, from the catalytic α subunit. The first class I RNRs, the class Ia enzymes, discovered and characterized more than 30 years ago, were found to use a diiron(III)-tyrosyl-radical Cys oxidant. Although class Ia RNRs have historically served as the model for understanding enzyme mechanism and function, more recently, remarkably diverse bioinorganic and radical cofactors have been discovered in class I RNRs from pathogenic microbes. These enzymes use alternative transition metal ions, such as manganese, or posttranslationally installed tyrosyl radicals for initiation of ribonucleotide reduction. Here we summarize the recent progress in discovery and characterization of novel class I RNR radical-initiating cofactors, their mechanisms of assembly, and how they might function in the context of the active class I holoenzyme complex.

In nearly all organisms, replication and repair of genetic material require transition metal ions, specifically *via* the action of ribonucleotide reductases (RNRs) (1, 2, 3). RNRs provide the only *de novo* source of 2′-deoxyribonucleotide building blocks for DNA biosynthesis by performing a chemically challenging dehydroxylation reaction upon a ribonucleotide di- or triphosphate substrate (4). The role of the transition metal cofactor in this process is to transiently oxidize an active site cysteine (Cys) side chain to a thiyl radical (Cys•) (2), which then initiates reaction with substrate by radical activation of the inert 3′-C-H bond in the ribose ring (Fig. 1*A*) (5). In this approach, an activating oxidation step at an adjacent site induces the desired cleavage of the neighboring 2′-C-O bond. At the end of the reaction, the thiyl radical is regenerated and the net reduction reaction is balanced by provision of reducing equivalents from two additional Cys residues in the active site or the external reductant, formate (6).

**Figure 1:**
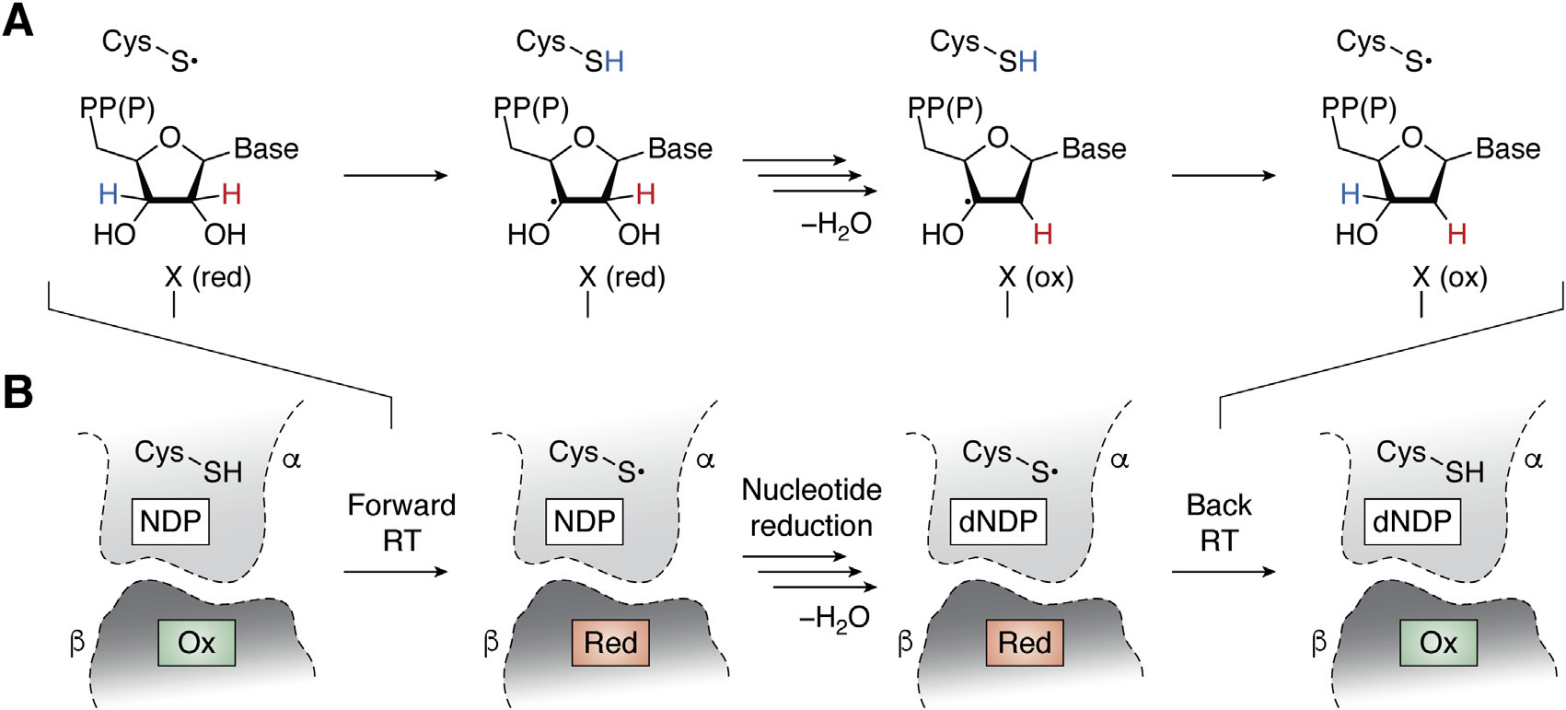
**Schematic of ribonucleotide reduction and radical translocation (RT) in class I RNR.***A*, universal mechanism for nucleotide reduction in RNRs. *B*, diagram of the steps involved in radical translocation in class I RNRs.

While all RNRs share this universal mechanism of ribonucleotide reduction, the metal-ion-driven method of thiyl radical generation varies substantially (2). The identity of the Cys oxidant provides the basis for classification of RNRs into three classes, I to III. Class II enzymes use a Co-dependent adenosylcobalamin cofactor to oxidize the active site Cys directly (7). Class III enzymes use an iron-dependent [4Fe-4S]-activase to generate a stable glycyl radical in the RNR active site (8), which then forms the thiyl radical locally. These enzymes are found exclusively in microbes and their metallocofactors are either agnostic to oxygen (class II) or potently inactivated by it (class III). By contrast, class I RNRs operate in all three domains of life, including humans and other eukaryotes (1, 2). Class I enzymes share a reliance on dioxygen for assembly of the Cys oxidant cofactor and a complex quaternary structure, in which the metallo- or radical cofactor is located in a separate enzyme subunit (9). This enzyme architecture necessitates long-range radical translocation (RT) for thiyl radical generation (Fig. 1*B*) (3, 10). The first class I RNRs to be characterized biochemically, class Ia enzymes, contain a diiron(III)-tyrosyl-radical (Fe_2_^III/III^-Y•) metallocofactor (Fig. 2) (11). More recent characterization of sequence-divergent class I RNRs has revealed remarkably varied inorganic chemistry associated with this enzyme class (2). Five class I RNR subclasses (class Ia–e) have now been reported (2, 12, 13, 14, 15, 16), four of which (Ib–e) deviate from the Fe_2_^III/III^-Y• cofactor of well-characterized Ia enzymes. The features that differentiate these five known class I RNRs include 1) the number and type of metal ions present in the active β subunit for cofactor maturation, 2) the oxidant used to generate the active cofactor (dioxygen or superoxide), 3) the involvement of additional activase proteins, and 4) the identity of the stable oxidant in β (metal- or Tyr•-based) that forms the Cys• in α (17). Here, in the context of the canonical class Ia RNR, we discuss progress in understanding cofactor assembly in Mn-dependent class Ib and Ic RNRs. We also summarize the discovery and characterization of the two most newly reported subclasses, Mn-dependent Id, and the apparently metal-free Ie enzymes. Finally, we present a bioinformatic analysis of the class I RNR β subunits, highlighting areas of sequence space that remain uncharacterized and might serve as fertile ground for discovery of other novel cofactors and their mechanisms of O_2_-mediated assembly.

**Figure 2:**
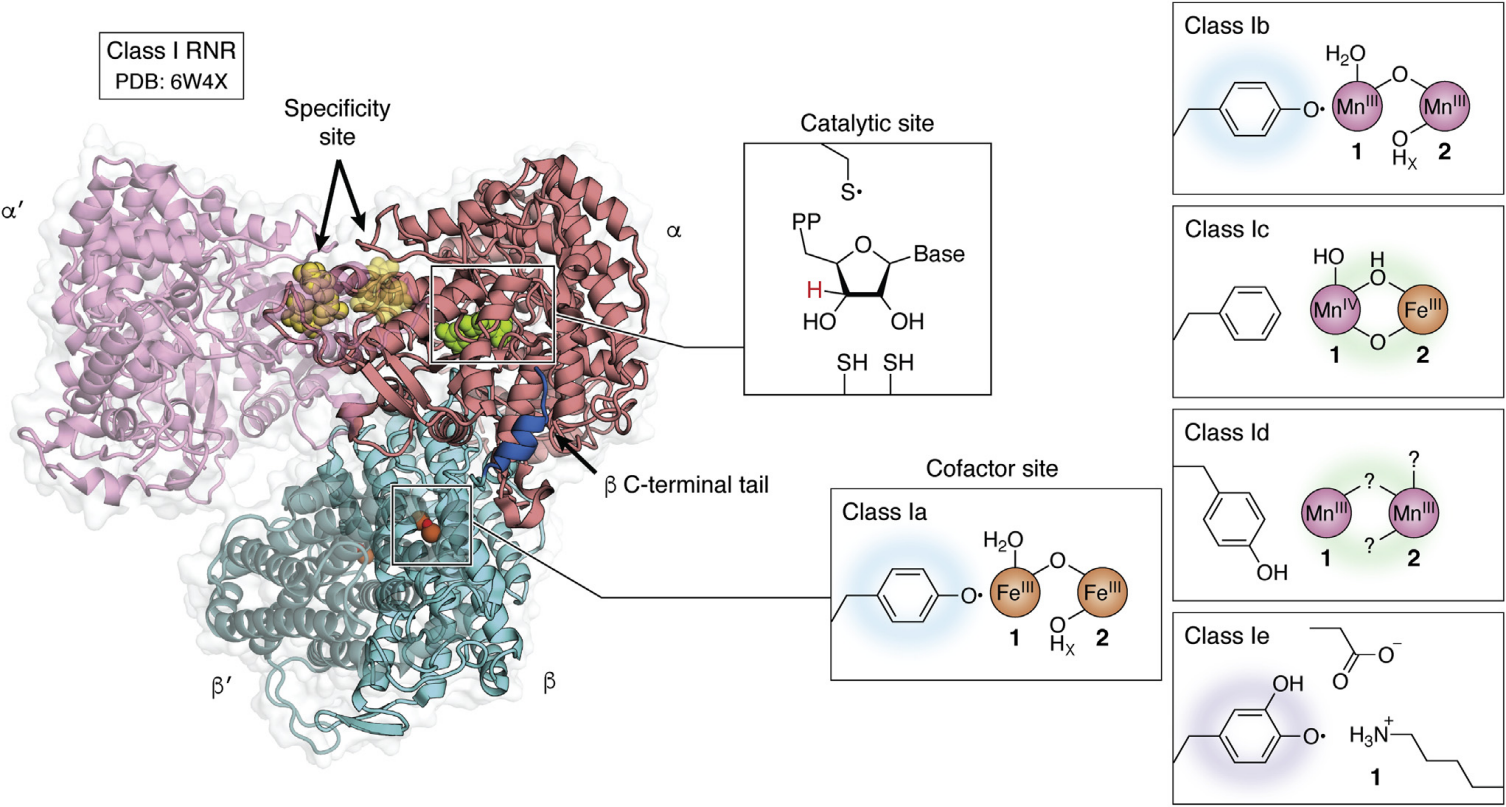
**Quaternary structure of the active holoenzyme complex in class I RNR (PDB accession code****6W4X****).***Insets* show the location of the active site in the catalytic α subunit (*middle top*) and the metallo- or radical cofactor (*middle bottom* and *far right*) in the β subunit.

## Fe-dependent class Ia RNR

**Figure 3:**
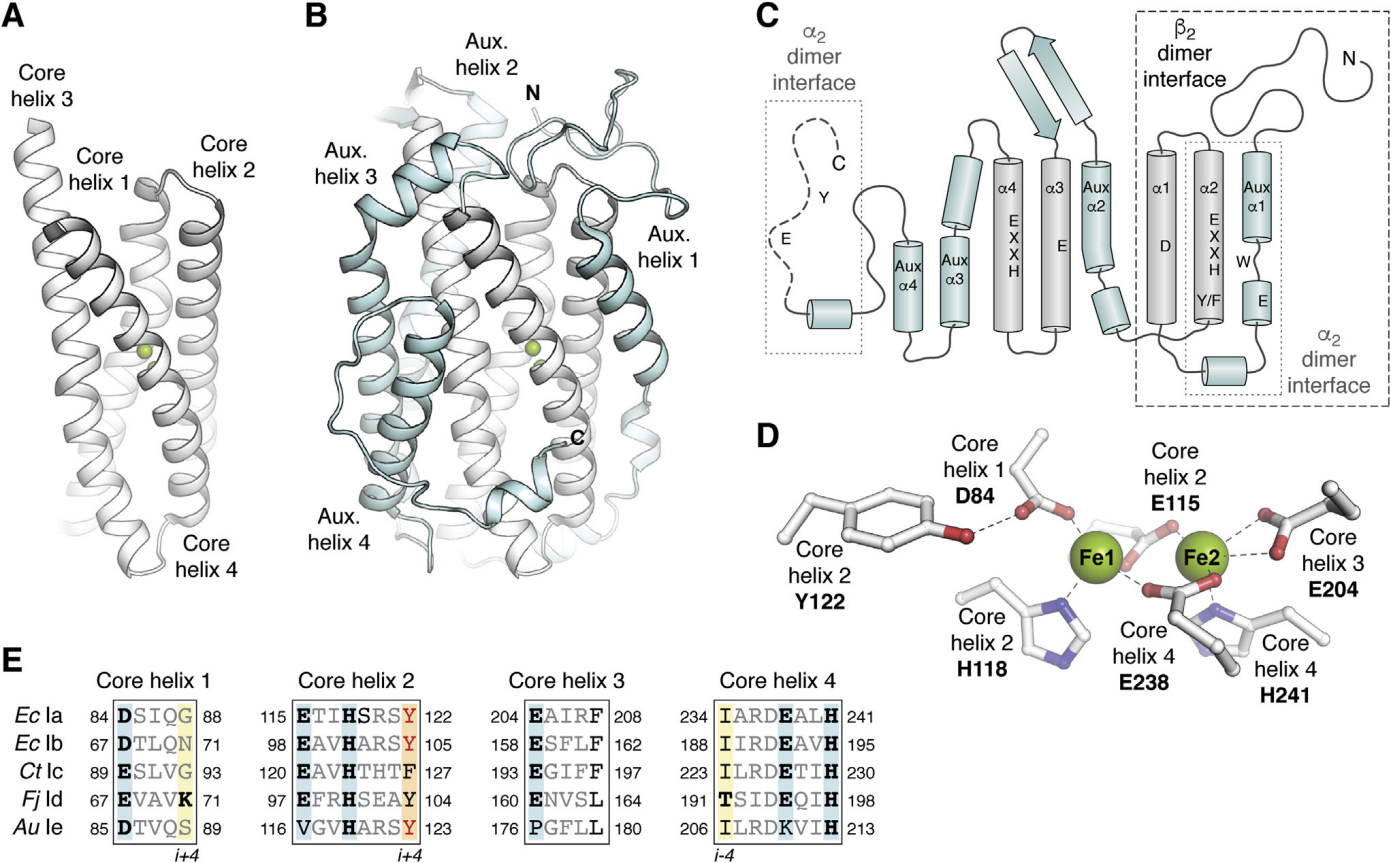
**Structural features of the class I RNR β subunits.***A*, core four-helix bundle implicated in metal binding and/or radical cofactor assembly (*white*) and *B*, auxiliary helices (*cyan*). *C*, topology diagram showing the locations of amino acids in class Ia RNR important for metal-binding, Tyr• formation, or radical translocation. *D*, metal-binding site in Fe^II^_2_-β of class Ia RNR (PDB accession code 1PIY). Selected amino acids shown in *stick* format and Fe^II^ ions shown as *green spheres*. Coordination interactions or hydrogen bonds shown as *dashed lines*. *E*, a sequence alignment showing conservation of important metal-binding (*cyan*) or radical formation sites (*orange*) in all known class I RNR subclasses. Amino acids important for oxidant access and suppression of Tyr• formation in class Id RNR are shown in *boldface* and highlighted in *yellow*.

Class I RNRs assemble a Cys oxidant cofactor in a β subunit. All β subunits characterized to date belong to the ferritin structural superfamily (9, 18), using a core four-helix-bundle motif to bind and assemble the active oxidant (Fig. 3, *A*–*C*). In class Ia RNRs, this core unit is symmetrical, with two pairs of α-helices arranged head-to-tail to fully bury the Fe_2_^III/III^-Y• oxidant and protect it from solvent. Each helix in the pair contributes a D/E or EXXH metal-binding motif (Fig. 3*D*). In addition to protection from solvent, the regular helical core structure also enables provision of second-sphere side chains that can contribute significantly to cofactor assembly and reactivity, often located one helical turn away from metal-binding side chains in *i* ± 3 or *i* ± 4 positions (Fig. 3*E*). For example, the tyrosine (Tyr) side chain that harbors the Y• in the activated form of class Ia RNR is located in the *i* + 4 position of core helix 2 relative to the EXXH iron-binding motif. Patterns of conservation in amino acids important for cofactor function can vary among class I RNR enzyme subclasses.

The core helical bundle of class I RNRs is surrounded by additional secondary structures, typically four auxiliary α-helices and flexible N- and C-termini (17, 18) (Fig. 3, *B* and *C*). These auxiliary structures are important for mediating interactions with other polypeptides. All class I RNRs rely on such interactions for catalysis (1, 2). The β subunit itself operates as a homodimer (Fig. 2), with an interface that involves the N-terminal half of the β-subunit-fold (9). In the most well-characterized class Ia RNR from *Escherichia coli*, the active holoenzyme adopts an α_2_β_2_ quaternary structure (Fig. 2) (19, 20) in which the α subunit contains the active site for nucleotide reduction and one or more allosteric regulatory nucleotide-binding sites (21). Formation of this complex involves N-terminal auxiliary secondary structures in β as well as the C-terminus of the protein (20). Interestingly, the final ∼30 amino acids of the β subunit are flexible and remain persistently disordered in X-ray crystal structures of β alone (9). Despite this disorder, the C-terminus of β forms a key part of the interface with the catalytic subunit and contains two amino acids essential for turnover, E350 and Y356 (*E. coli* Ia numbering) (20).

The primary function of the α-β subunit interface in class I RNRs is reversible translocation of an oxidizing equivalent, or radical, from the cofactor in β to the active site in the α subunit (3, 10) (Fig. 1*B*). This process occurs with remarkable speed, up to 10 s^−1^ in the fastest class I enzymes (22), and over a great distance, estimated to span a ∼35 to 40 Å distance in docking models of the two subunits (21, 23). The distance between oxidant source and destination requires involvement of aromatic amino acids in RT (10). Conserved Tyr and tryptophan (Trp) amino acids, spanning both subunits, were initially implicated as these intermediates based on conservation and requirement for activity (21, 24). Initiation of RT and formation of these pathway intermediates must be tightly controlled. For example, the oxidizing equivalent in β is only deployed once substrate is bound to the α subunit (25). This step and subsequent Tyr oxidation steps are proposed to involve proton-coupled electron transfer (PCET) events (10). In *E. coli* class Ia RNR, the entire radical translocation process is gated by a slow conformational change within the holoenzyme complex (22, 25), and this feature prevents direct detection of pathway radical intermediates in the wild-type enzyme. To overcome this issue, key Tyr side chains in *E. coli* class Ia RNR have been successfully substituted with noncanonical analogs of altered reduction potential (26, 27). These substitutions slow down the radical transfer steps such that they are no longer masked by conformational change, allowing for observation of pathway Tyr• at sites such as Y356 in the C-terminus of β (28). Recent advances in cryo-electron microscopy technology, together with insight from experiments to perturb radical translocation, enabled capture of the structure of the active form of the *E. coli* class Ia RNR holoenzyme complex just last year (20). Here, we also summarize the key findings of this tour-de-force study and its implications for understanding radical translocation in other class I RNR subclasses.

**Figure 4:**
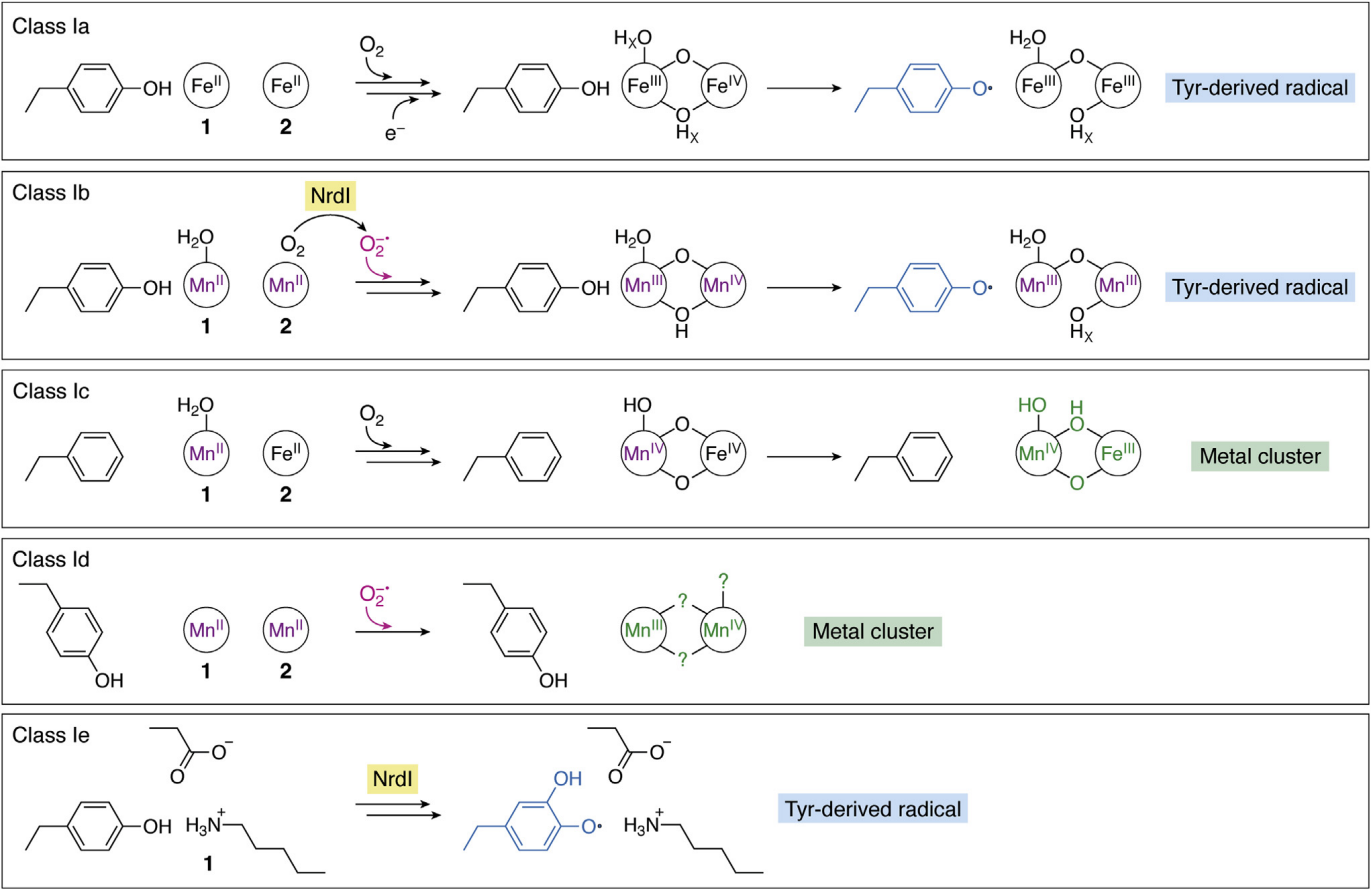
**Cofactor assembly mechanisms for class I RNRs.** Manganese-dependent enzymes are highlighted in *purple*. Superoxide-dependent RNRs are highlighted in *hot pink*. Subclasses that require an NrdI activase are indicated with a *yellow box*. Metal-centered Cys oxidants shown in *green* and Tyr-derived radical Cys oxidants shown in *blue*.

Assembly of the active cofactor in the β subunit is an important maturation step in class I RNR catalysis, one that likely provides additional opportunities for regulation of enzyme activity (2). Cofactor maturation is best understood in the Fe_2_^III/III^-Y•-utilizing *E. coli* class Ia RNR (Fig. 4). In this enzyme, the β subunit can be activated *in vitro* in a self-assembly reaction that only requires purified apo β protein, Fe^II^, oxygen, and a reductant. Inside the cell, this process probably involves other components, including factors that control metallation and cofactor oxidation state (29). The former step is important because mismetallation with other metal ions, such as Mn^II^, occurs readily and potently inhibits the enzyme (30). During the *in vitro* self-assembly reaction, apo β loads with two equivalents of Fe^II^, and this form of the protein has been structurally characterized (31). The dinuclear cofactor is coordinated by the four carboxylate and two histidine ligands contributed by the four-helix core of the ferritin fold (Fig. 3*D*) (31). The Tyr122 side chain that ultimately becomes oxidized to a Y• in the activated cofactor resides near one of the metal ions, Fe1. The metal-binding site is fully buried from solvent but an internal hydrophobic cavity and adjacent open coordination positions suggest an access route for oxygen (Fig. 3*D*) (31, 32). The next step in the cofactor maturation reaction is rapid addition of O_2_ to the Fe_2_^II/II^ cluster, yielding a short-lived peroxo-Fe_2_^III/III^ intermediate (33, 34). A conserved second-sphere aromatic, Trp48, donates an electron to reductively cleave the peroxide unit forming a Fe_2_^III/IV^ intermediate, **X** (35, 36). The resulting Trp48^+•^ is reduced by the necessary external reducing agent (36). Intermediate **X** oxidizes the adjacent Tyr122 to form the Fe_2_^III/III^-Y• cofactor (Fig. 4) (35, 36), capable of initiating RT to the α subunit to oxidize the active site Cys. Forward RT results in a Fe_2_^III/III^ state that can reversibly reform the active Fe_2_^III/III^-Y• after nucleotide reduction is completed (Fig. 1*B*) (2). The RT product complex is likely distinct from a separate Fe_2_^III/III^ (met) form of the active cofactor that forms *via* unproductive 1-electron reduction of the Y•. The difference between these two cluster forms could involve subtle changes in cofactor structure and/or protonation state. The ferritin fold and its buried cofactor site insulate against proton transfer in and out of the interior of the protein, allowing for control of RT initiation *via* carefully controlled PCET steps. The met Ia cofactor is stable and conversion back to its active form requires reduction of the Fe^III^ ions (37).

## Recent advances in class Ia RNR holoenzyme characterization

The active metallo- or radical cofactor in class I RNR β subunits is ultimately used to generate a transient thiyl radical in the α subunit to initiate the ribonucleotide reduction reaction (1). This intersubunit radical translocation (RT) is triggered in the β subunit upon substrate binding to the active site in α. RT occurs on every turnover of the enzyme, and it is reversible, with the oxidizing equivalent returning to the β subunit after the reaction is finished. Docking models and pulse electron paramagnetic resonance (EPR) measurements estimated an RT distance of 35 to 40 Å for this process (21, 23, 38). To account for the rate of class I RNR turnover (∼1–10 s^−1^), which is too fast for single-step electron transfer, RT is proposed to involve smaller proton-coupled electron transfer steps mediated by conserved aromatic amino acid side chains arranged in a pathway between the cofactors in each subunit (2). In the past decade, a series of elegant studies by Stubbe and coworkers (10, 27, 28) have established functional roles for conserved aromatics along the RT pathway in *E. coli* class Ia RNR by showing that modulation of reduction potentials of pathway Tyr *via* incorporation of noncanonical amino acid analogs can slow RT sufficiently to detect transient radical intermediates localized to these sites (10).

**Figure 5:**
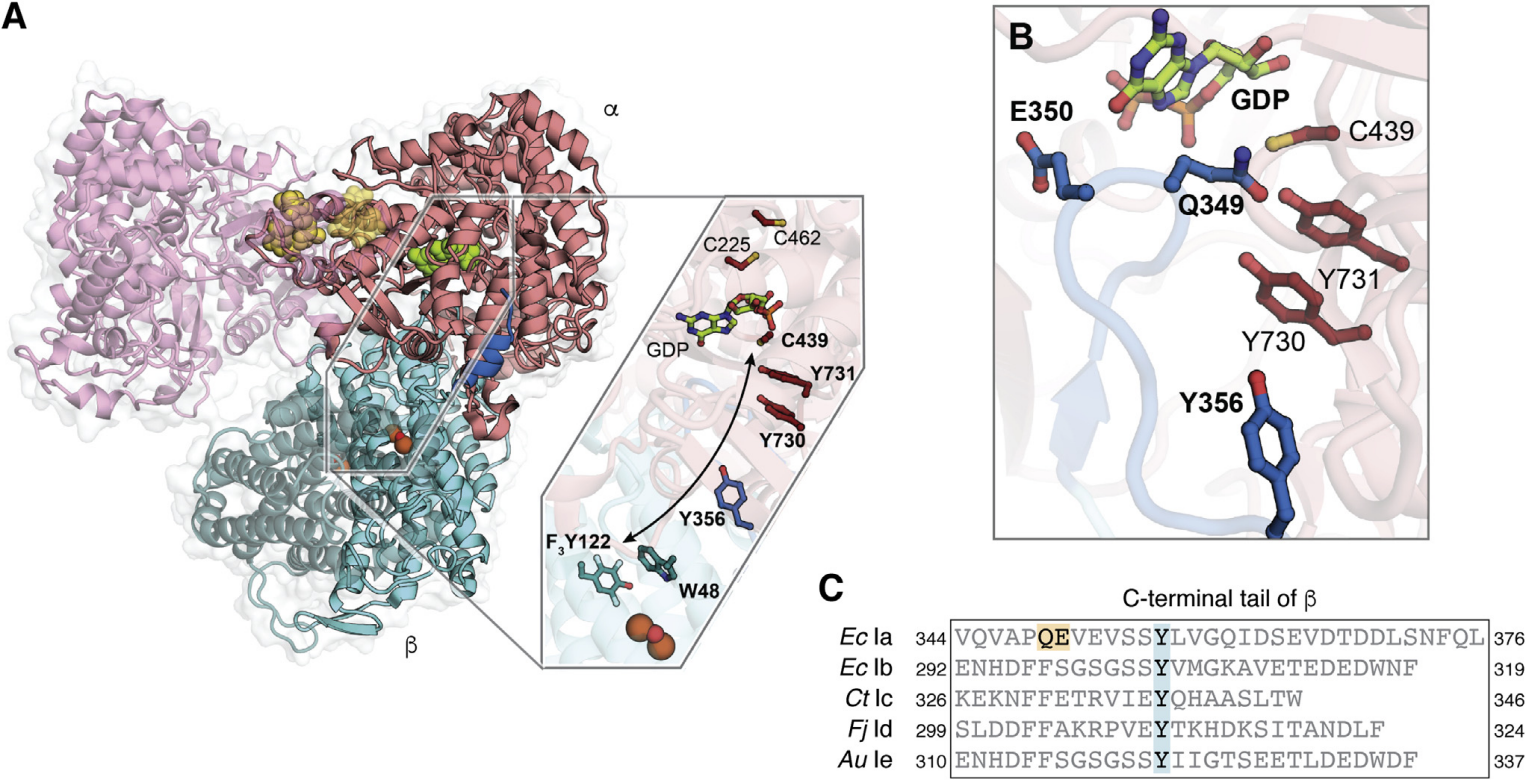
**Structure of the active holoenzyme complex in class Ia RNR (PDB accession code****6W4X****).***A*, the engaged half of the holoenzyme complex reveals the arrangement of aromatic amino acid side chains along the RT pathway (indicated by *arrow* and *boldface* labels). *B*, a zoomed-in view of the ordered C-terminus of the β subunit shows important interactions with the GDP substrate. Panels *A* and *B* adapted from refs. (3, 20). *C*, a sequence alignment of the β subunit C-terminal tail in representative members of class Ia–e subclasses. RT pathway intermediate Y356 is conserved among all class I RNRs but the polar side chains implicated in substrate binding in class Ia RNR differ in Ib–e RNRs.

The capacity for rapid and reversible electron transfer across the subunit interface has inspired a great deal of interest in the structure of the active holoenzyme complex in any class Ia RNR. Efforts to obtain this information in the *E. coli* Ia system have been hindered by the dynamic nature of subunit interaction (39) and a propensity to form allosterically inhibited higher-order structures at high protein concentration (19). For example, the *k*_off_ for α-β dissociation is estimated to be >60 s^−1^ (39). However, some of the modifications used to characterize pathway Tyr radicals also stabilize the holoenzyme complex for several minutes (40). In combination with technical advances in cryo-electron microscopy approaches, which allow for sample analysis on the seconds-to-minutes timescale at low protein concentration, Stubbe, Drennan, and coworkers recently reported a 3.6-Å-resolution structure of the *E. coli* class Ia α_2_β_2_ holoenzyme complex by using a E52Q/(2,3,5)-F_3_-Y122-substituted version of β (20) (Fig. 5*A*). This structure significantly advances our understanding of class I RNR radical translocation by showing how the active complex assembles to arrange PCET components upon substrate binding and disassembles to permit product release. Here we review aspects that are likely to be conserved among all class I RNRs and contrast those with the details that might differ in class Ib–e enzymes, thereby warranting further study.

One of the most striking features of the *E. coli* class Ia RNR active holoenzyme complex is its asymmetry (20) (Fig. 5*A*). Only one half of the subunit interface engages at a time. Therefore, the RT pathway cannot simultaneously assemble in both halves of the α_2_β_2_ complex. This result is consistent with experimental observations of half-of-sites reactivity in class Ia RNRs (41). Other subclasses also exhibit evidence of this phenomenon. In class Ib RNR, a low-resolution X-ray crystal structure of an α_2_β_2_ complex shows a similar asymmetric intersubunit interaction (42). And in class Ic RNR, detection of two kinetic phases of hydroxyurea-(HU)-mediated cofactor reduction during enzyme turnover supports half-of-sites reactivity and complex asymmetry in this subclass (43). The fast phase is interpreted as HU-mediated interception of a pathway Tyr• in the engaged half of the holoenzyme while a slow phase represents direct reaction of HU with the Mn^IV^Fe^III^ cofactor in the other subunit. While half-of-sites reactivity has not been investigated to date in class Id or class Ie RNR, class Ie enzymes exhibit ∼50% sequence identity to class Ib enzymes, suggesting they likely engage the α subunit similarly.

All class I RNRs characterized to date conserve a Tyr residue (Y356, *E. coli* Ia numbering) in a C-terminal tail of the β subunit that is typically disordered in X-ray structures of the β_2_ complex alone (2). This residue is essential for RNR activity because it mediates RT between the two subunits of the holoenzyme complex, but its functional location had never been visualized previously. On one side of the *E. coli* class Ia holoenzyme complex, the entire C-terminus orders to reveal the location of Y356 and all other residues in this previously disordered tail (20). The side chain of Y356 is found adjacent to a pair of conserved Tyr side chains in the α subunit, also implicated in PCET, consistent with an RT mediator role for Y356 (Fig. 5*A*). Interestingly, Y356 does not appear to have a dedicated amino acid proton donor/acceptor. Instead, water molecules at the subunit interface are implicated in this process, although these waters cannot be detected directly in the cryo-EM structure. While all class I RNRs conserve a Y356 equivalent, we cannot yet infer its exact structural context, including proximity to interfacial water molecules, in other subclasses.

The ordering of the β subunit C-terminus revealed by the cryo-EM structure of the *E. coli* class Ia holoenzyme complex also shows conformational changes that could communicate substrate binding in the α subunit to the Cys oxidant in the β subunit (20). The C-terminus of the β subunit folds into the active site of the catalytic subunit to make both direct and indirect contacts to substrate (Fig. 5*B*). This observation shows that the β subunit detects the status of the active site (substrate, product, nothing bound) directly *via* ordering of the β subunit C-terminal tail. This mechanism of intersubunit communication also explains the dynamic nature of subunit interaction. Because the tail blocks the active site, the complex must fully dissociate to release product and rebind substrate. Interestingly, the two key amino acids in the β subunit C-terminus that contact substrate in *E. coli* class Ia RNR, Q349 and E350, are not universally conserved in other subclasses (Fig. 5*C*), suggesting differences in the exact structure of the C-terminus of the β subunit may exist in other RNRs that use alternative cofactors.

These conformational changes are likely an important part of the event (or events) that initiate radical translocation from the β subunit cofactor upon substrate binding in α. The mechanism of RT initiation still remains elusive due to the low local resolution of the cryo-EM structure in the vicinity of the metallocofactor (∼5.5 Å resolution) and the nature of the mutations needed to trap a stable complex (20). Analysis of the α subunit active site structure suggests that the disengaged half of the holoenzyme complex has undergone a single turnover, due to observation of a disulfide bond in that subunit. The other half of the complex appears to be organized for radical translocation but the oxidizing equivalent in β has not yet deployed to the α subunit. The link between the substitutions made in this system and the structural observations remains unclear—but the effects on Y122 reduction potential and p*K*_a_ conferred by the fluoro groups of the (2,3,5)-F_3_ modification are likely the key perturbations dictating outcome (27, 44). Two other recent studies of class Ia and Ic RNR provide some clues about the molecular details of RT initiation in class I enzymes. Mössbauer analysis of the RT product in *E. coli* class Ia RNR suggests that Fe1-mediated proton transfer to the Y• at position 122 involving a terminal water ligand is the first step in oxidant deployment (45). And the aforementioned study of HU-induced cofactor reduction in class Ic RNR concludes that the key chemical event responsible for Cys oxidant excursion occurs in the β subunit and local to the cofactor (43). Interestingly, these proposals would indicate that the chemical details of the first step of radical translocation in other subclasses could differ substantially. The metal-centered oxidants of class Ic and Id enzymes lack the Tyr• proton destination (12, 14) and class Ie enzymes lack a metal-coordinated water molecule in their active forms (15, 16).

Iron-dependent class Ia RNRs are dominant in eukaryotes, including humans (46), and widespread in bacteria (47). Among prokaryotes, acquisition of iron to drive DNA replication can present a challenge in nutrient limited environments (48). To adapt, other RNR subclasses have evolved in bacterial and archaeal organisms employing different metallocofactors and/or radical components (2, 3). These distinctions necessitate novel mechanisms of cofactor maturation (Fig. 4), including requirement for additional proteins or alternative O_2_-derived metal/Tyr oxidants. The next sections of this review focus on the alternative metallo- or radical cofactors found among class I RNRs, with an emphasis on subclasses that use transition metal ions other than iron and their cofactor assembly mechanisms. Efforts to understand these aspects of RNR catalysis are significant because Ib–e enzymes dominate in aerobic bacterial pathogens. Evolution of new Cys oxidants likely provides a competitive advantage against human and animal hosts that rely on iron-dependent RNRs.

## Mn-dependent class Ib RNR

**Figure 6:**
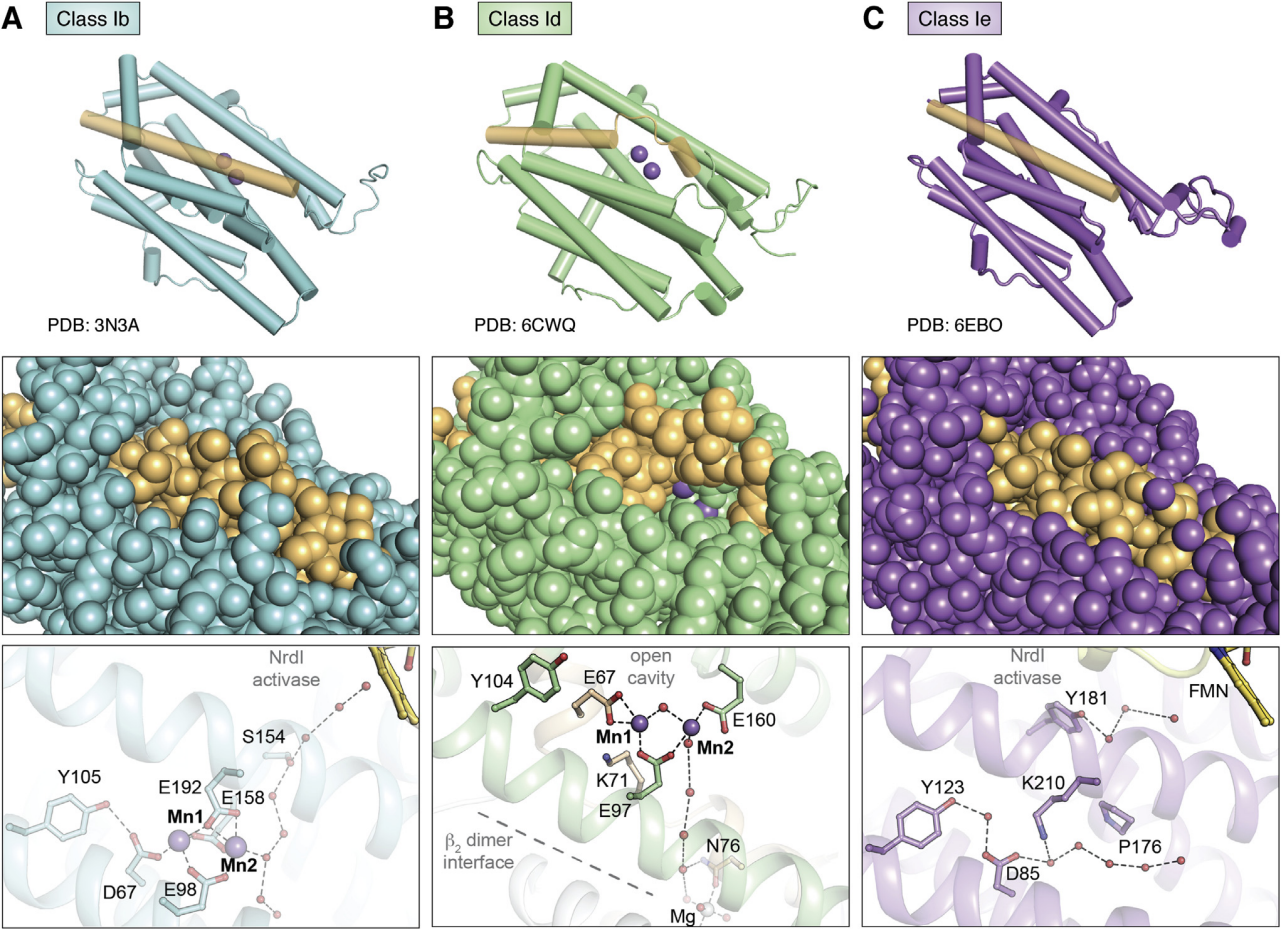
**Comparison of overall structure (*top*), cofactor solvent exposure (*middle*), and location of solvent channels for superoxide access (*bottom*) in class Ib (*A*) (PDB accession code****3N3A****), class Id (*B*) (PDB accession code****6CWQ****), and class Ie RNR (*C*) (PDB accession code****6EBO****).** Selected amino acids shown in *stick* format and Mn^II^ ions shown as *purple spheres*. Coordination interactions or hydrogen bonds shown as *dashed lines*. Water molecules shown as *red spheres*.

Class Ib RNRs were first differentiated from class Ia enzymes on the basis of sequence identity (49). Despite early indications of a reliance upon manganese for activity (50), particularly in enzymes isolated from their native host organisms (51, 52), this subclass was initially proposed to assemble an Fe_2_^III/III^-Y• (53). This form is active *in vitro* (13, 54), albeit to a lesser extent than typical class Ia RNRs (22). The importance of manganese in class Ib RNR function was definitively established in 2010 by Cotruvo and Stubbe (13). A highly active Mn_2_^III/III^-Y• can be formed by reconstitution of Ib β with Mn^II^, oxygen, and the reduced form of a flavoprotein, NrdI (13) (Fig. 4). Consistent with an essential role in active cofactor assembly, NrdI is universally encoded in the genomes of class-Ib-utilizing organisms, typically as part of the same operon as the Ib α and β subunits. The activase binds to the Ib β subunit, an interaction that has been characterized crystallographically in two different class Ib homologs (32, 55). The activation complex contains a hydrophilic channel that links the flavin cofactor in NrdI to the Mn_2_^II/II^ site in the β subunit (Fig. 6*A*) (32). This structural feature shows a feasible route to Mn-dependent class Ib RNR cofactor assembly involving flavin-mediated remote generation of a reduced form of O_2_ (superoxide or peroxide) that is channeled within the activation complex to oxidize a reduced dimanganese cluster. In this model, the function of the universally encoded NrdI activase is to overcome the inherent inertness of O_2_ to reaction with Mn^II^ by providing a more reactive form of the cofactor oxidant. This model also represents a rare example of use of normally toxic reactive oxygen species to perform a beneficial function inside the cell.

More recently, several different research groups have pursued open questions associated with the biological significance of class Ib Mn-dependent RNR activity, the detailed mechanism of Mn_2_^III/III^-Y• cofactor assembly and its optimization, and the structural and mechanistic diversity of class Ib RNRs. The observation of significant *in vitro* activity with either iron or manganese in these systems led to a series of investigations into which form is predominantly used in native organisms. In 2011, two different groups investigated class Ib RNR usage in *E. coli*, a facultative anaerobe encoding two aerobic RNRs, class Ia and Ib (56, 57). Martin and Imlay demonstrated that Ib RNR is only expressed upon derepression of its operon by the Fe^II^-responsive transcription factor, Fur (56). This observation is consistent with a biological role for class Ib RNR in enabling cell replication when iron levels are low. Cotruvo and Stubbe reached a similar conclusion, showing that class Ib RNR isolated from *E. coli* strains deficient in five different iron uptake systems contains only manganese (57). Importantly, this work represented the first isolation and characterization of the active cofactor in an Ib RNR obtained without overexpression. Class Ib RNRs have also been isolated with active Mn_2_^III/III^-Y• cofactors from homologous overexpression systems in two different native organisms (*Cornyebacterium ammoniagenes* and *Bacillus subtilis*) that rely on the Ib enzyme as their sole aerobic source of deoxyribonucleotides (51, 52, 58). The active enzyme from *C. ammoniagenes* was characterized comprehensively by EPR spectroscopy and X-ray crystallography (52), providing the most complete description to date of an activated Ib RNR. Metal ion usage by class Ib RNR has also been investigated in the opportunistic pathogen, *Streptococcus sanguinis*, which encodes an Ib enzyme as its sole aerobic RNR (59, 60). In a rabbit model of *S. sanguinis*-mediated infective endocarditis, the gene encoding NrdI, required for Mn-dependent Ib activity but not iron-dependent activity, was shown to be essential for growth and survival of the pathogen (60). A recent transcriptomics study of *S. sanguinis* response to Mn-depletion under aerobic conditions shows downregulation of the Ib operon accompanied by diminished growth (61). These results are consistent with inability of the *S. sanguinis* Ib RNR to substitute iron for manganese *in vivo*.

In 2013, a kinetic dissection of the metallocofactor assembly in *B. subtilis* class Ib RNR provided important information about the identity of the Mn^II^ oxidant generated by NrdI and a key intermediate responsible for Tyr oxidation (Fig. 4) (62). Stopped-flow absorption spectroscopy analysis of NrdI flavin oxidation showed that only the two-electron-reduced, NrdI_hq_, form of the activase reacts with O_2_ quickly enough to account for the overall rate of cofactor assembly. This observation implicates a single equivalent of superoxide as the Mn^II^ oxidant. Rapid freeze-quench EPR (RFQ-EPR) spectroscopy analysis of the first observable oxidized metal-centered intermediate in this system reveals a spectrum assigned as a Mn^III^Mn^IV^ cluster. This species decays at a rate that matches that of Tyr• appearance, as measured in the stopped-flow absorption analysis. Provision of a single equivalent of superoxide for cofactor assembly is both a creative and economical solution to class Ib cofactor assembly (62). Not only does it generate a form of O_2_ that reacts readily with Mn^II^, but it also provides the exact number of oxidizing equivalents required to generate two Mn^III^ ions and a Tyr•.

Mechanistic studies of class Ib RNRs have been hindered by diversity in yields of active cofactor and differences in structure, β subunit affinity, and redox properties of the NrdI activases. The divergence of biochemical properties is perhaps not surprising because phylogenetic analysis of NrdI sequences reveals three distinct groups of Ib enzymes (63). The structurally well-characterized *E. coli* and *C. ammoniagenes* Ib systems belong to a group that consistently yields low levels (0.2–0.3 Tyr•/β) of active cofactor upon isolation or *in vitro* reconstitution (13, 52). NrdIs from a second phylogenetic group, including proteins from *B. subtilis* and related organisms, exhibit variable redox potentials for different forms of the flavin cofactor. While *E. coli* and *B. subtilis* NrdIs cannot stabilize significant quantities of one-electron-reduced NrdI_sq_ (54, 62), *Bacillus anthracis* NrdI can stabilize quantitative amounts of the semiquinone, similar to traditional flavodoxins (64). Based on this observation, maturation of *B. anthracis* Mn_2_^II/II^-β has been proposed to initiate from a NrdI_sq_ state. However, detailed kinetic analysis of the assembly mechanism, as performed for *B. subtilis* class Ib RNR, would be required to definitively establish this distinction. In 2014, Lofstad *et al.* (65) reported an important advance in enhancing levels of Tyr• in *Bacillus cereus* NrdI-dependent cofactor assembly by screening candidate flavodoxin reductases for those that enhance yield of active cofactor. The approach produced 0.6 Tyr• per β, a threefold improvement in yield over initial reconstitutions in the absence of a reductase. Application of this tactic to other class Ib enzymes could facilitate mechanistic study and biophysical characterization of active Mn cofactors or oxidized intermediates.

## Mn- and Fe-dependent class Ic RNR

The class Ic enzyme from *Chlamydia trachomatis* was the first class I RNR shown to use a metal ion other than iron in its active cofactor (12). It was also the first class I RNR demonstrated to use a metal-based Cys oxidant rather than a Tyr•-derived oxidant. In 2007, Jiang *et al.* showed that maximal enzyme activity could be achieved with *in vitro* cofactor assembly in the presence of Mn^II^ and Fe^II^ (Fig. 4). The active cofactor is a Mn^IV^Fe^III^ complex, formed by reaction of a Mn^II^Fe^II^ precursor with O_2_. By an alternative mechanism, the *C. trachomatis* RNR can also be activated by reaction of Mn^II^Fe^II^ with two equivalents of H_2_O_2_ (66). Both the Mn^IV^Fe^III^ state and a preceding Mn^IV^Fe^IV^ intermediate have been extensively characterized by Mössbauer, EPR, and X-ray absorption spectroscopy (XAS) (67). These efforts were instrumental in establishing the identities and oxidation/spin states of these complexes. Recently, researchers have focused on understanding additional structural features of the class Ic active cofactor and its assembly pathway.

Crystallographic characterization and application of advanced spectroscopic techniques to the Mn-bound forms of class Ic RNR have enabled detailed assignment of metal ion and proton location in the active cofactor and its precursors (68, 69, 70). Two different crystallographic analyses of activated Ic enzymes revealed that the Mn^IV^ ion resides in site 1 (68, 70), the metal-binding site closest to the Tyr• in class Ia/b enzymes (Fig. 4). This assignment is consistent with functional substitution of the redox-active Tyr by an oxidized Mn ion in the class Ic enzymes. The Mn site 1 assignment was also corroborated by spectroscopic studies on activated enzyme samples in solution (69). The crystallographic studies further revealed partial occupancy of site 1 by iron and site 2 by manganese, particularly when the enzyme was reconstituted with excess of either metal ion (68, 70). This finding is consistent with a known propensity for mismetallation of this scaffold.

**Figure 7:**
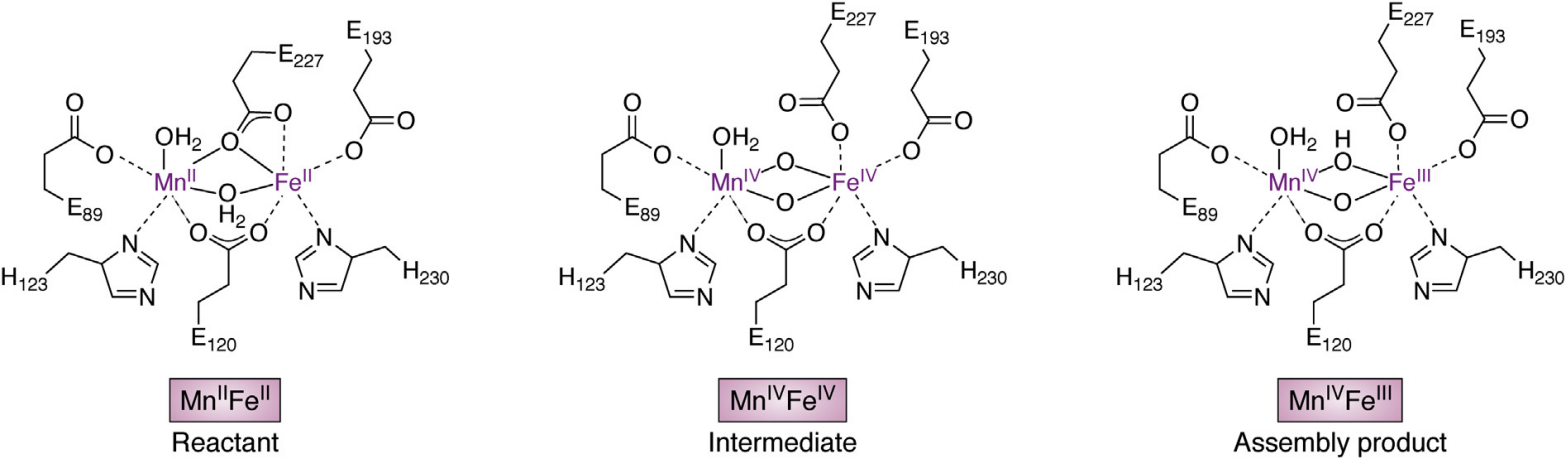
**Structures of reactant (*left*), intermediate (*middle*), and assembly product (*right*) in Mn**^**IV**^**Fe**^**III**^**cofactor activation in class Ic RNR**.

In a subsequent crystallographic study of the reactant Mn^II^Fe^II^ complex, issues with site-selective metal ion binding were overcome *via* extended anaerobic incubation of apo class Ic RNR β with Mn^II^ and Fe^II^ (71). In this experiment, each metal ion was present in 1:1 ratio per β monomer, resulting in strict partitioning of Mn to site 1 and Fe to site 2. Interestingly, structures of apo β co-crystallized with Mn^II^ show occupancy of both sites, even at substoichiometric metallation. Selective assembly of the relevant precursor to the active cofactor might be driven, therefore, by a preference of iron for site 2 in class Ic RNR. Interestingly, analyses of metal ion binding in *E. coli* class Ia RNR are also consistent with higher affinity of iron for site 2 (72, 73). The structure of Mn^II^Fe^II^-β additionally provides insight into the details of the first coordination sphere prior to O_2_ addition (71). The reactant state contains two water ligands, one bound terminally to Mn1 and another that bridges the two metal ions (Fig. 7). The structure also reveals a μ-η^1^,η^2^ binding mode for one of two bridging Glu ligands, an interaction seen in the fully reduced forms of other dimetal-binding class I RNR β subunits. Interestingly, this ligand undergoes significant conformational change upon cofactor oxidation, the sole protein ligand to do so in class Ic RNR.

The relatively small number of conformational and coordination changes upon conversion to the active form of class Ic RNR have made it possible to fully map the structures of intermediate and product states *via* spectroscopic and computational approaches (Fig. 7). XAS and pulse EPR spectroscopic studies of the Mn^IV^Fe^IV^ intermediate in class Ic RNR are consistent with a di-μ-oxo bridging structure and a terminal Mn^IV^ hydroxide ligand (74), suggesting that O_2_ inserts between the metal ions to displace the μ-η^1^,η^2^-bridging Glu ligand, accompanied by deprotonation of the solvent ligand to Mn1. A recent valence-to-core X-ray emission study of this intermediate and the active Mn^IV^Fe^III^ cofactor confirms this assignment for the Mn^IV^Fe^IV^ complex (75). Spectral comparisons show elongation of one Mn-O bond concomitant with contraction of the other upon 1-electron reduction, consistent with protonation of one of the two oxo bridges. The structure of the further reduced Mn^III^Fe^III^ product of the RT reaction remains to be defined in detail, but it may be accompanied by similar proton transfers to the active cofactor, a phenomenon that is probably universal to reaction initiation in all class I RNRs.

## Mn-dependent class Id RNR

Class Id ribonucleotide reductases were initially distinguished from previously characterized class I RNRs by a unique Tyr/Glu pairing in the first and second coordination sphere, as opposed to a Tyr/Asp pairing in Ia/Ib and Phe/Glu pairing in Ic β (Fig. 3*E*) (14). In the biochemical characterization of Id β from *Flavobacterium johnsoniae*, Rose *et al.* demonstrated that efficient activation of metal-depleted Id β occurs in the presence of Mn^II^, O_2_ and superoxide-generating compounds, such as naphthoquinol or hydroquinol. Cofactor assembly is inhibited by the presence of the O_2_^−^ scavenger, superoxide dismutase, confirming the ability of Id β to utilize environmental sources of O_2_^−^. Using a combination of XAS, EPR, and UV–visible spectroscopy experiments, the active cofactor was confirmed as a stable Mn_2_^III/IV^ cluster. Even though the class Id RNRs universally conserve a second-sphere Tyr in the location that harbors a radical in class Ia and Ib enzymes, *F. johnsoniae* Id RNR does not oxidize this side chain to form a stable Tyr• during cofactor assembly. The oxidized Mn_2_^III/IV^ cluster serves as the radical initiator in this system, making Id enzymes the second example of a metal ion-based Cys oxidant after the heterodimeric Mn^IV^Fe^III^ cofactor of Ic RNR (14). Two other class Id RNR β subunits from *Facklamia ignava* and *Leeuwenhoekiella blandensis* have been characterized in their activated forms by EPR, yielding similar spectral features to those of Mn_2_^III/IV^ *F. johnsoniae* β (76, 77)

Class Id RNR cofactor assembly bears some resemblance to class Ib cofactor maturation, the only other solely Mn-dependent class I RNR identified to date (62). The class Id cofactor assembly mechanism is arguably simpler because it requires only the addition of Mn^II^ and O_2_^−^ to metal-depleted preparations of β. Class Ib RNR cofactor assembly also involves superoxide-dependent oxidation of a di-manganese cluster, but the metal ion oxidant is generated by the flavoprotein activase, NrdI (13), generally encoded adjacent to the Ib α and β subunits. Of the >260 Id RNR β sequences identified to date, none are found encoded near an NrdI (14). Additionally, Id β can be activated *in vitro* independent of an accessory activase. Class Ib enzymes also share the ability to achieve a Mn_2_^III/IV^ oxidation state, but only as a transient intermediate, which then decays to a Mn_2_^III/III^ state concomitant with Tyr• formation (62). Interestingly, the active Id cofactor easily converts back to a Mn_2_^II/II^ state poised for reactivation, *via* cooperative three-electron reduction of the Mn_2_^III/IV^ complex (14). This behavior contrasts with the reductive decay pathways of class Ia–c cofactors (2). In these systems, the one-electron reduced metal ion clusters (Fe_2_^III/III^, Mn_2_^III/III^, or Mn^III^Fe^III^) are inert to further reduction and likely require a dedicated reductase inside the cell to reactivate the enzyme.

**Figure 8:**
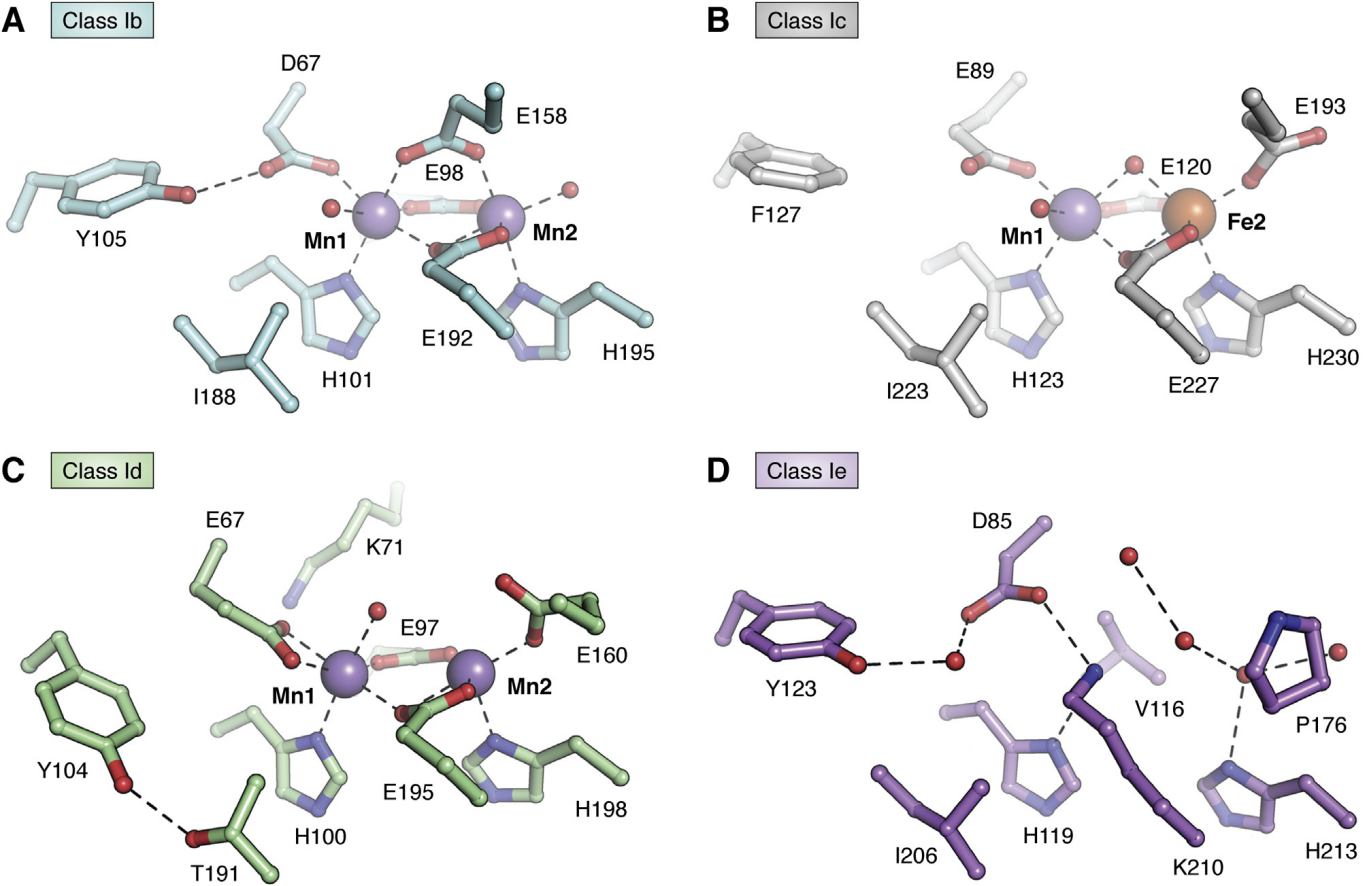
**Comparison of metal-binding sites in class Ib (*A*) (PDB accession code****3N3A****), class Ic (*B*) (PDB accession code****4M1I****), class Id (*C*) (PDB accession code****6CWP****), and class Ie RNR (*D*) (PDB accession code****6EBO****).** Selected amino acids shown in *stick* format. Mn^II^ ions shown as *purple spheres*, Fe^II^ ions shown as *orange spheres*. Coordination interactions or hydrogen bonds shown as *dashed lines*. Water molecules shown as *red spheres*.

Structural characterization of class Id Mn_2_^II/II^-β subunits has provided insight into the structural basis for direct superoxide scavenging and use of a metal ion-based Cys oxidant. Id β from *F. johnsoniae* was the first enzyme in this subclass to be structurally characterized in a defined metal ion-bound form, revealing a solvent-exposed metal center resulting from an unwinding of core helix 1 and a distinctive orientation for the second-sphere Tyr (Fig. 6*B* and 8, *A*–*C*) (14). The helical distortion occurs immediately after Mn1 ligand Glu67 (*F. johnsoniae* numbering), exposing a ∼300 Å^3^ cavity and the metal cluster to solvent (14). This distortion and cavity have also been observed in metal ion-bound structures of *L. blandensis* and *F. ignava* β (76, 77). In the former system, the structural deviation seems to be the result of metal ion loading because the corresponding helix is uninterrupted in apo structures of *L. blandensis* β (77). A conserved positively charged side chain, lysine (Lys71) (*F. johnsoniae* numbering), within the unwound portion of helix 1 resides within the vicinity of the metal center—the first incidence of a positive charge adjacent to a dimetal center in any class I β (Fig. 8) (14). Rose *et al.*, proposed that Lys71 and the open cavity function in electrostatic attraction of negatively charged superoxide.

Tyr104 (*F. johnsoniae* numbering), the Tyr•-harboring residue in Ia and Ib RNR, possesses a novel hydrogen bonding partner, Thr191, in class Id enzymes (Fig. 8*C*). This interaction sequesters Tyr104 ∼7 Å away from Mn1. By contrast, the corresponding Tyr in class Ia and Ib RNR is positioned much closer (∼4–5 Å) to the metal cluster and in H-bonding contact with a unique Asp ligand (Figs. 3*D* and 8, *A* and *C*) (31, 32). These interactions could facilitate Tyr oxidation, whereas the alternative orientation and interactions in Id RNR are likely linked to the inability to form and stabilize a Tyr• in activated Id β. Surprisingly, the Tyr104 side chain remains essential for RNR activity, playing an as-yet unknown role in the radical translocation process (14). When compared with class Ia–c RNRs, the significant structural differences associated with the reactant Mn_2_^II/II^ form of the β subunit in class Id enzymes underscore the distinctive cofactor assembly and maintenance pathway utilized by this subclass.

Certain class Id β subunits have additional functional domains appended to their N-terminus. *F. ignava* and *L. blandensis* Id β subunits contain ATP cones (77), an overall activity regulatory domain typically found at the N-terminus of the α subunit in class Ia and Ic RNRs (1). In class Ia RNR, ATP-binding to the ATP cone promotes formation of active α_2_ dimers with exposed β_2_ binding sites while dATP association stabilizes inhibited higher-order oligomers incapable of functional interaction with β_2_ (19). All class Id RNR α subunits identified to date lack an ATP cone domain (78), but this regulatory unit is found instead in selected β subunits (77). In X-ray crystal structures of ATP-cone bearing *L. blandensis* β, the subunits form tetramers when bound to dATP. While this quaternary structure superficially resembles the inactivated α_4_ structures observed in dATP-bound *E. coli* class Ia RNR (19), the mechanism of ATP-cone-mediated β subunit inhibition remains unclear. The class Id β_4_ tetramer forms exclusively *via* ATP cone interactions, leaving the predicted α binding site unobstructed (77). Nucleotide-independent regulation of the α subunit activity may also be possible in Id systems lacking ATP cones entirely (78). *F. johnsoniae* α can form tetramers in solution, but the physiological relevance of these structures and implications for activity are open questions.

## Metal-free class Ie RNR

Class Ie RNRs were codiscovered in 2018 by two independent research teams (15, 16). The Ie β subunit sequences are similar in overall sequence identity to class Ib enzymes, but with a striking substitution of three metal ion coordinating Glu side chains (Figs. 3*E* and 8*D*) (15, 16, 79). Interestingly, RNR β subunits with this property were first reported by Roca *et al.* (79), 10 years earlier. In this study, two Ib-like RNR operons were identified within the *Streptococcus pyogenes* genome, each encoding a catalytic α subunit, a β subunit, and an NrdI homolog. One contained a true Ib β with the anticipated D…EXXH…E…EXXH metal-binding motif while the other encoded a β sequence with the aforementioned substitutions at three of six metal-coordinating Glu residues (Fig. 3*E*). The components of the latter operon showed no activity *in vitro* but could complement an *E. coli* strain possessing a temperature-sensitive class Ia RNR mutation (79). This early study did not, however, identify the Cys oxidant assembled in the unusual β subunit. More recently, these experiments were replicated with *Aerococcus urinae* and *Mesoplasma florum* Ie homologs, showing that each is capable of nucleotide reduction in *E. coli*, provided that all three components (α, β, NrdI) were coexpressed (15, 16). Bioinformatic analyses uncovered more than 400 bacterial RNR β sequences with the characteristic substitutions of Ie enzymes, some of which represent the sole aerobic RNR encoded in the organism’s genome (15). Together, these observations suggest that class Ie RNRs are capable of ribonucleotide reduction activity despite the significant changes to the predicted metal-binding motif.

Although involvement of an NrdI activase in cofactor assembly is a shared feature with class Ib β subunits, the identity of the class Ie radical-initiating cofactor distinguishes these enzymes from other class I RNR subclasses (15, 16). Activated β subunits can be isolated from NrdI coexpression cultures for the *S. pyogenes*, *A. urinae*, and *M. florum* Ie homologs. These preparations yield active enzyme in *in vitro* activity assays (0.18–0.35 s^−1^ per β subunit), but the β subunit contains <0.2 equivalents of any first-row transition metal. X-ray crystallography, EPR, and mass spectrometry analysis of activated Ie β subunits revealed that the active Cys oxidant is a 3,4-dihydroxyphenylalanine radical (DOPA•), generated *via* posttranslational modification of Tyr123, the site of Tyr• formation in class Ia/b RNRs. X-ray structures of activated Ie β subunits show no evidence of a transition metal component, leading to the conclusion that the active form of β is metal ion-free. Interestingly, a conserved Lys residue (Figs. 3*E*, 6*C* and 8*D*) appears to substitute metal site 1, suggesting that Ie enzymes may use this alternative cation as a surrogate for the oxidized metals of other class I RNR active cofactors.

The identity of the Ie radical initiator is surprising because the predicted redox potential of an anionic DOPA• (∼0.6 V) would be inadequate to oxidize a Cys side chain (∼0.9 V) (28). To illustrate this point, semisynthetic incorporation of DOPA on the protein surface at the position of Tyr356 in *E. coli* Ia β results in a trapped DOPA• radical and an inactive enzyme (28). However, pulse EPR analysis of activated class Ie β established that the buried DOPA• in this system is instead found in a neutral form (15). We propose that the DOPA• redox potential is tuned by its protonation state and surrounding protein environment to render it a sufficiently potent oxidant for the Cys.

Class Ie RNRs have not yet been successfully reconstituted *in vitro* from purified components. All active preparations of Ie β studied to date were generated by coexpression with NrdI inside the cell (15, 16). A full understanding of the components and conditions necessary for DOPA• maturation remains to be elucidated. Once these factors are defined, we can begin to understand the order of events in cofactor maturation—radical generation *versus* Tyr hydroxylation—and maintenance. Srinivas *et al.* (16), showed that after reductive inactivation of DOPA• by HU, ∼10% of the inactivated enzyme could be reconstituted *via* redox cycling in the presence of NrdI and O_2_, suggesting that it is possible to form active radical from DOPA *via* the action of NrdI alone. However, the exact mechanism of the reactivation and its implications for DOPA• maturation from Tyr are not known. We also do not understand the conditions in which class Ie RNR is used inside the cell, although a metal-free radical initiating cofactor would seem advantageous for pathogens susceptible to iron or manganese limitation induced by the host immune system. In support of this hypothesis, Do *et al.* (80) demonstrated that increased intracellular Fe and Mn represses Ie RNR expression in *S. pyogenes*, an organism that encodes both a Mn-dependent class Ib RNR and a Ie system. This observation would be consistent with a role for the Ie enzyme as a backup or failsafe for DNA replication when trace metal ions are scare. Work to more fully decipher the conditions in which Ie RNRs are expressed in native organisms could reveal additional cofactor maturation requirements and information about the biological significance of this unusual RNR subclass.

## Bioinformatic analysis of class I RNR β subunits

**Figure 9:**
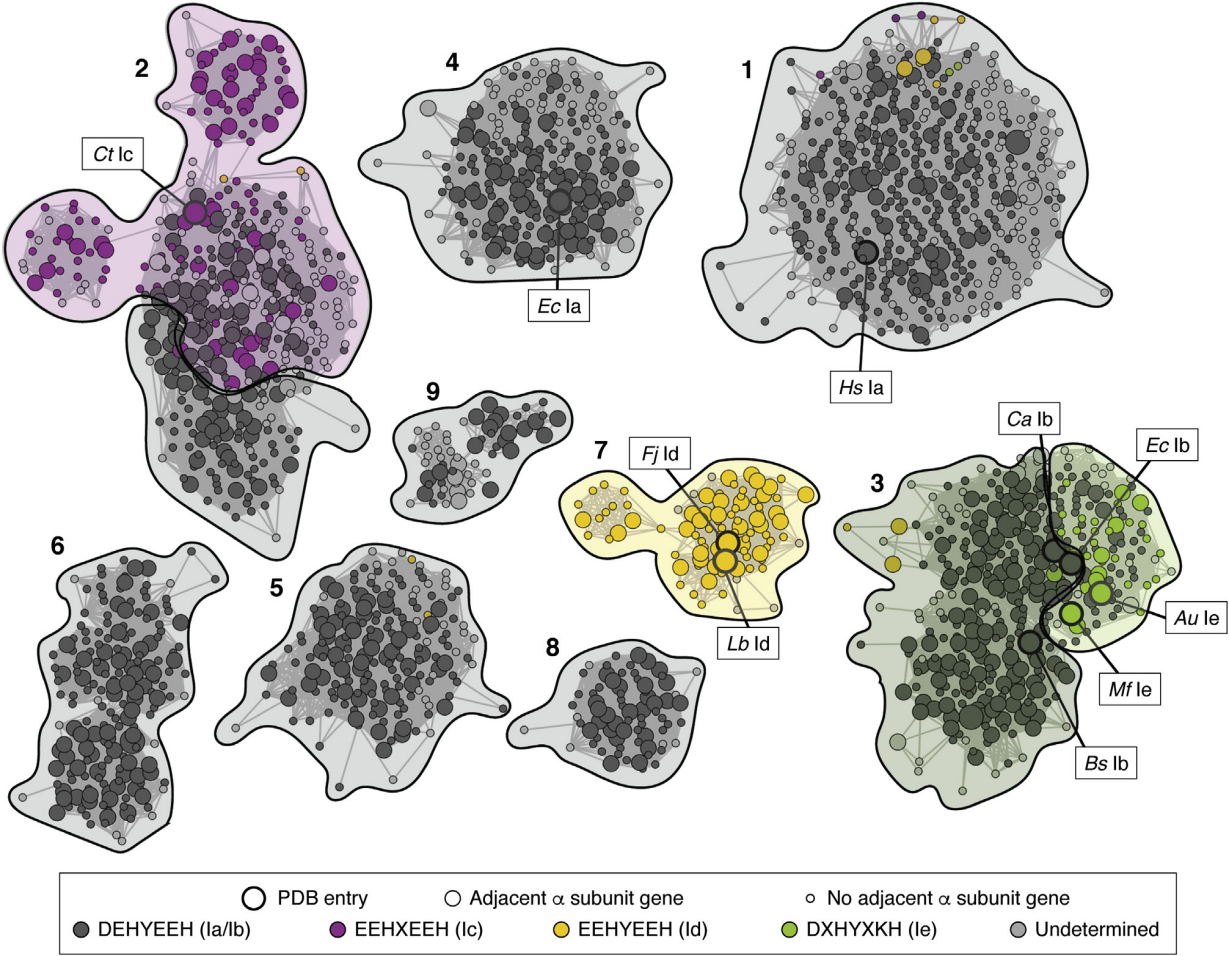
**A sequence similarity network (SSN) (alignment score = 80, nodes represent sequences that share >50% identity) showing the top nine clusters, ranked by number of nodes, for annotated class I RNR β subunits.** All clusters have a significant number of β subunits with an adjacent catalytic subunit encoded. Nodes are colored by conservation of subclass-specific metal-binding motif or conservation of the radical-harboring Tyr. Outlined nodes denote sequences characterized structurally. Labeled nodes indicate sequences with experimentally validated cofactor assignments discussed in this review. *Shading* indicates subclass assignment of a sequence cluster based on assessments of sequence and genomic context.

Sequence comparisons have often provided crucial initial clues about use of novel cofactors and assembly pathways in class I RNR β subunits. A map of sequence space for annotated class I RNR β proteins highlights the areas in which cofactor usage and biochemical properties remain to be explored. Figure 9 shows the top nine clusters (ranked by number of nodes, in which each node represents a set of sequences with >50% identity) in a sequence similarity network (SSN) (81) generated by analysis of >31,000 annotated class I RNR β sequences (82) extracted from UniProtKB (September 2019 release). We annotated the SSN by genome neighbor patterns (node size) and conservation of predicted metal-binding residues (node color). Enlarged nodes indicate a neighboring class I RNR α catalytic subunit verified in the genome. Node color indicates conservation of an Ia/b-like (dark gray), a Ic-like (purple), a Id-like (yellow), or a Ie-like metal-binding motif (light green). Clusters are shaded according to the dominant subclass present in that cluster. Note that at the current alignment score of 80, this SSN groups sequences with Ic-like metal binding together with Ia-like homologs, and Ib-like sequences are found together with Ie-like counterparts (indicated as purple/gray shading in cluster 2, dark green/light green shading in cluster 3). Biochemically characterized class I RNR β subunits discussed in this review are indicated as boxes.

Class Ia-like sequences can be found in clusters 1, 2, 4, 5 to 6, and 8 to 9. Of these sequence clusters, only cluster 4 contains a comprehensively characterized enzyme (*E. coli* Ia RNR) in terms of cofactor assembly, stability, and holoenzyme complex structure. This cluster predominately contains other closely related proteobacterial sequences. The other sequence groups are largely unexplored even though they contain eukaryotic and viral RNRs (cluster 1) and enzymes from important bacterial pathogens such as *Pseudomonas aeruginosa* (cluster 2), *Campylobacter jejuni* (cluster 6), and *Anaplasma phagocytophilum* (cluster 5). Clusters 8 and 9 are dominated by environmental actinobacterial and bacteriophage sequences, respectively. These untapped areas of class I β subunit sequence space might harbor unexpected bioinorganic and/or radical chemistry that could be exploited in development of novel drugs that target class I RNRs.

## Future directions and outlook

The reliance, for the most part, of ribonucleotide reduction on transition metal ions provides a window into how organisms adapt to changing availability of trace elements in the environment or in complex consortia of microbes and their hosts. Among class I RNRs, the discovery of alternative metal-ion- or radical-based cofactors for DNA biosynthesis has led to elucidation of novel biochemistry associated with cofactor assembly and radical translocation. Important research objectives for the future include (1) continued mining of class I RNR sequence space for novel cofactors; (2) investigation of holoenzyme complex structures beyond the *E. coli* class Ia RNR model system; (3) understanding pathways of cofactor assembly, regulation, and maintenance inside the cell; and (4) exploration of the evolutionary relationships among class I RNR subclasses.

## Conflict of interest

The authors declare no conflicts of interest with the contents of this article.
